# Postglacial range expansion and the role of ecological factors in driving adaptive evolution of *Musa basjoo* var. *formosana*

**DOI:** 10.1038/s41598-017-05256-6

**Published:** 2017-07-13

**Authors:** Jui-Hung Chen, Chun-Lin Huang, Yu-Long Lai, Chung-Te Chang, Pei-Chun Liao, Shih-Ying Hwang, Chih-Wen Sun

**Affiliations:** 1 Department of Life Science, National Taiwan Normal University, 88 Tingchow Road, Section 4, Taipei, 11677 Taiwan; 2 Laboratory of Molecular Phylogenetics, Department of Biology, National Museum of Natural Science, 1 Guanchien Road, Taichung, 40453 Taiwan; 3 Department of Geography, National Taiwan University, 1 Roosevelt Road, Section 4, Taipei, 10617 Taiwan

## Abstract

Genetic variation evolves during postglacial range expansion of a species and is important for adapting to varied environmental conditions. It is crucial for the future survival of a species. We investigate the nuclear DNA sequence variation to provide evidence of postglacial range expansion of *Musa basjoo* var. *formosana*, a wild banana species, and test for adaptive evolution of amplified fragment length polymorphic (AFLP) loci underlying local adaptation in association with environmental variables. Postglacial range expansion was suggested by phylogeographical analyses based on sequence variation of the second intron of copper zinc superoxide dismutase 2 gene. Two glacial refugia were inferred by the average *F*_ST_ parameter (mean *F*_ST_ of a population against the remaining populations). Using variation partitioning by redundancy analysis, we found a significant amount of explained AFLP variation attributed to environmental and spatially-structured environmental effects. By combining genome scan methods and multiple univariate logistic regression, four AFLP loci were found to be strongly associated with environmental variables, including temperature, precipitation, soil moisture, wet days, and surface coverage activity representing vegetation greenness. These environmental variables may have played various roles as ecological drivers for adaptive evolution of *M. basjoo* var. *formosana* during range expansion after the last glacial maximum.

## Introduction

In the Quaternary, temperature oscillations are an important historical factor influencing the current distributions of a plant species^1^. During the last glacial maximum (LGM), most plant species in the Northern hemisphere would have retreated southward toward the tropics or warmer lowland areas, and survived in refugia^2^. Taiwan is a continental island situated off the coast of the Asian mainland and lies to the south of the Ryukyu Arc and north of the Philippines Archipelago. Although remnants of glaciations at the top of some peaks along the central mountain range (CMR) were found^3^, the lowlands of Taiwan were not covered with ice but were drier and colder, and the climate changes would have confined species to refugia in low elevations^3, 4^. In Taiwan, many conifers escaped to the middle elevations during the LGM^4^. These species were originally distributed at middle and low elevations and would have migrated to the lowlands. A reverse course of events occurred since the LGM^1, 2, 5^, with species that were previously confined to refugia in the south expanded polewards and lowland forests colonizing at higher elevations^1, 2, 6^. Environmental gradients can potentially act as selective drivers during postglacial range expansion and result in locally adapted variants^2^. The current distributions of species are the results of a combinatorial effect by historical events, ecological factors, and stochastic or neutral mechanisms.

Population adaptive divergence is a central issue in evolutionary biology that focuses on understanding the correlations of population genetic variation with environmental heterogeneity^7^. Studies have shown that natural population divergence is driven by variable environmental conditions and leads to the evolution of locally adapted lineages^8–11^. However, gene flow between closely related genetic lineages can be reduced by a combinatorial effect of geography and environment; and geographical isolation may play a larger role than environmental variation in shaping population structure^11–13^. Therefore, investigating the relative roles of geography and environment that influence genetic variation is critical to understand how environmental factors may act as selective drivers and lead to adaptive genetic variation underlying local adaptation in natural populations of a species^11, 12^. Moreover, identifying environmental factors that play roles in driving adaptive divergence is particularly of interest in biological conservation and ecological restoration^14^.

In Taiwan, genetic signatures of postglacial expansion into ranges of habitats from refugia were observed in many tree species such as *Cyclobalanopsis glauca* ^15^, *Cunninghamia konishii* ^16^, *Trochodendron aralioides* ^17^, *Castanopsis carlesii* ^18^, and *Cinnamomum kanehirae* ^6, 19^. Although Taiwan spans a small range of latitude geographically, varied geographical topologies support vegetation from tropical to cool climates^20^. The dramatic topological differences combined with the influence of tropical and subtropical climates have fostered high habitat diversity and may serve as a driving force for adaptive evolution^9–12^. During the postglacial recolonization process, a species would have evolved with adaptive variation invoked by differential selection along environmental gradients occurring in species’ distribution ranges.

*Musa basjoo* Siebold is a cold hardy banana species and is thought to be originated from southern China^21, 22^ and is genetically differentiated from other *Musa* species^22, 23^. *M. basjoo* Siebold & Zucc. ex linuma var. *formosana* (Warb. ex Schum.) Ying is a variety of *M. basjoo* endemic to Taiwan^24^. *M. basjoo* var. *formosana* is distributed in low elevations in Taiwan^25^ and has a diploid chromosome number of 2n = 22^26^. The generation time of bananas under cultivation can be up to 18 months^25^ and could be longer for natural populations of *M. basjoo* var. *formosana*. Fruit from *M. basjoo* var. *formosana* is edible but has numerous large and hard seeds. This species is an important germplasm for banana breeding due to its characteristics of cold tolerance and disease resistance. For conservation of this species, it is important to identify the genetic relationships of individuals among populations and to understand the potential for evolutionary adaptation because biodiversity is increasingly threatened by human-induced anthropogenic climate change^27^.

**Figure 1:**
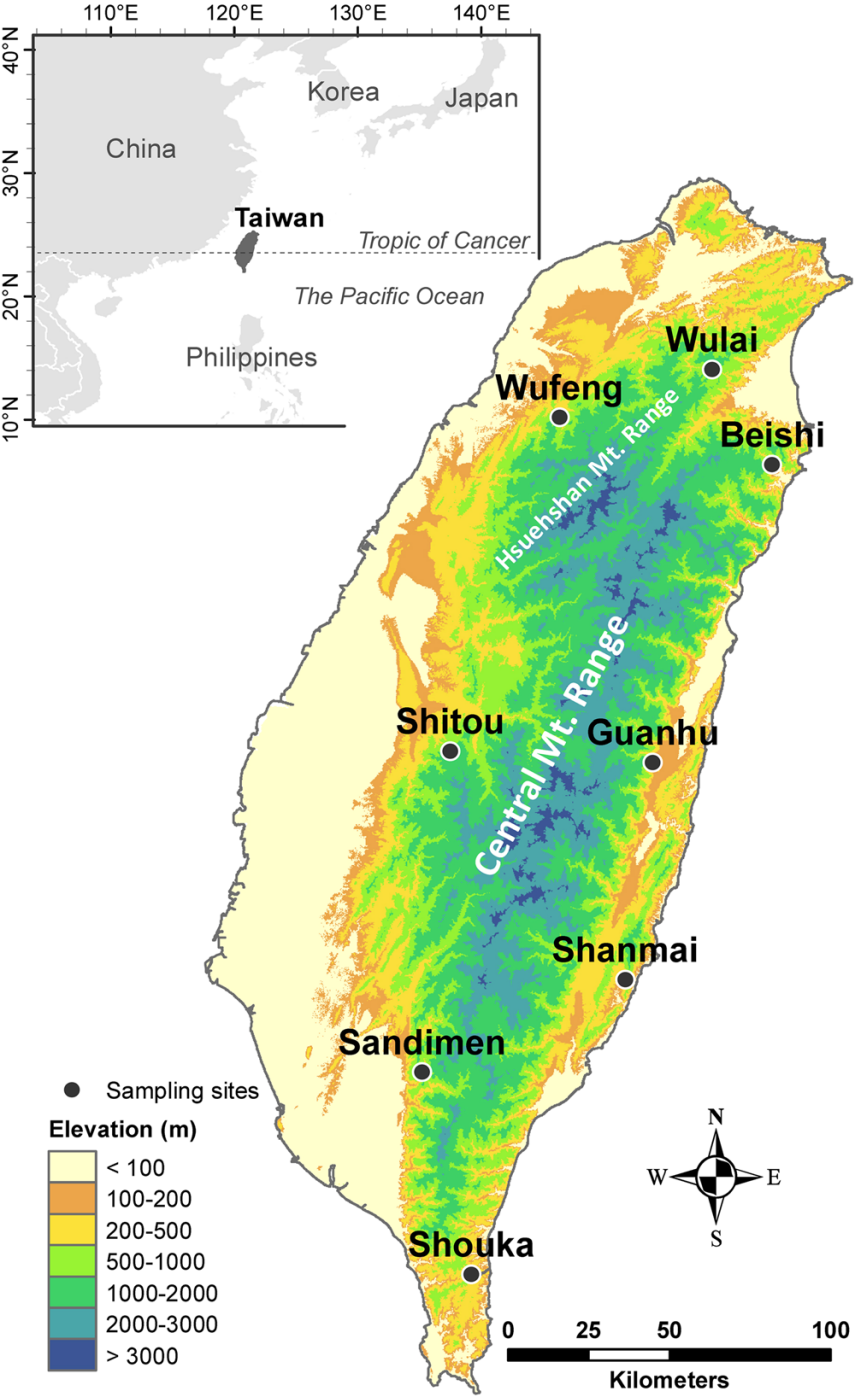
Sample localities of eight populations of *Musa basjoo* var. *formosana*. The countries’ boundary (polygon) map was derived from the default map database in ArcGIS v. 10.3^92^. The elevation gradients of Taiwan (background) were presented in ArcGIS based on the 20 m digital elevation model (DEM) that was acquired from Data.GOV.TW^93^. The locations of the sampling sites were plotted using Tools in ArcGIS by their coordinates.

In this study, the second intron of copper zinc superoxide dismutase 2 gene (Cu/Zn *SOD2*) was sequenced for 46 individuals from eight populations (Table 1 and Fig. 1) to characterize the postglacial recolonization event. Moreover, amplified fragment length polymorphism (AFLP)^28^ was also used to survey genetic variation of 112 individuals to examine the genetic relationships of individuals from different populations and test for association of AFLP loci with environmental variables underlying local adaptation driven by environmental gradients. We used variation partitioning based on redundancy analysis (RDA)^29^ to assess the relative influences of geographical and ecological isolation contributes to AFLP variation. *F*_ST_ outliers were identified using genome scan methods. In light of understanding local adaptations associated with environmental gradients, multiple univariate logistic regression was used to correlate environmental variables with AFLP loci that have potentially evolved under selection. We hypothesized that frequencies of AFLP outliers may display correlations with environmental gradients underlying local adaptation because the distribution of *M*. *basjoo* var. *formosana* in different geographical regions represents habitat environmental heterogeneities across the CMR. The main goals of this study were to: (i) demonstrate postglacial expansion of *M*. *basjoo* var. *formosana*; and (ii) find evidence for potential adaptive evolution associated with environmental gradients.

**Table 1:** Population, localities, and descriptive statistics of *Musa basjoo* var. *formosana* based on the second intron sequences of Cu/Zn SOD2.

Population	Locality (latitude/longitude)	Elevation (m)	No. of sequences	Haplotypes^a^	*Hd* (SD)	*θ*_π_ (SD)	*θ*_S_ (SD)
Beishi	24**°**30′/121**°**45′	441	12	I (6), Id (2), IIc (2), IId (2)	0.727 (0.109)	0.00173 (0.00038)	0.00157 (0.00064)
Guanhu	23**°**40′/121**°**23′	763	12	I (8), Ig (2), IIb (2)	0.545 (0.144)	0.00096 (0.00030)	0.00105 (0.00052)
Sandimen	22**°**48′/120**°**41′	406	12	I (12)	0	0	0
Shanmai	23**°**03′/121**°**17′	303	10	I (4), Ic (2), Ij (2), II (2)	0.800 (0.089)	0.00141 (0.00035)	0.00140 (0.00063)
Shitou	23**°**42′/120**°**46′	937	12	I (4), Ia (2), Ib (2), Ih (2), II (2)	0.848 (0.067)	0.00144 (0.00032)	0.00157 (0.00064)
Shouka	22**°**14′/120**°**50′	324	12	I (9), II (3)	0.409 (0.133)	0.00097 (0.00032)	0.00079 (0.00045)
Wufeng	24**°**38′/121**°**06′	489	10	I (4), Ie (1), If (1), Ii (2), IIa (2)	0.822 (0.097)	0.00157 (0.00036)	0.00168 (0.00069)
Wulai	24**°**51′/121**°**34′	645	12	I (8), II (4)	0.485 (0.106)	0.00115 (0.00025)	0.00079 (0.00045)
Total			92	16	0.628 (0.056)	0.00119 (0.00014)	0.00280 (0.00066)

*Hd*, haplotype diversity; *θ*_π_, nucleotide diversity estimated based on the average pairwise number of differences between sequences, *θ*_S_, nucleotide diversity estimated based on the number of segregating sites per sequence.

^a^The numbers in parentheses indicate the frequency of haplotype.

## Results

### Nucleotide diversity, population differentiation, and test for postglacial expansion based on the second intron sequences of Cu/Zn *SOD2*

**Figure 2:**
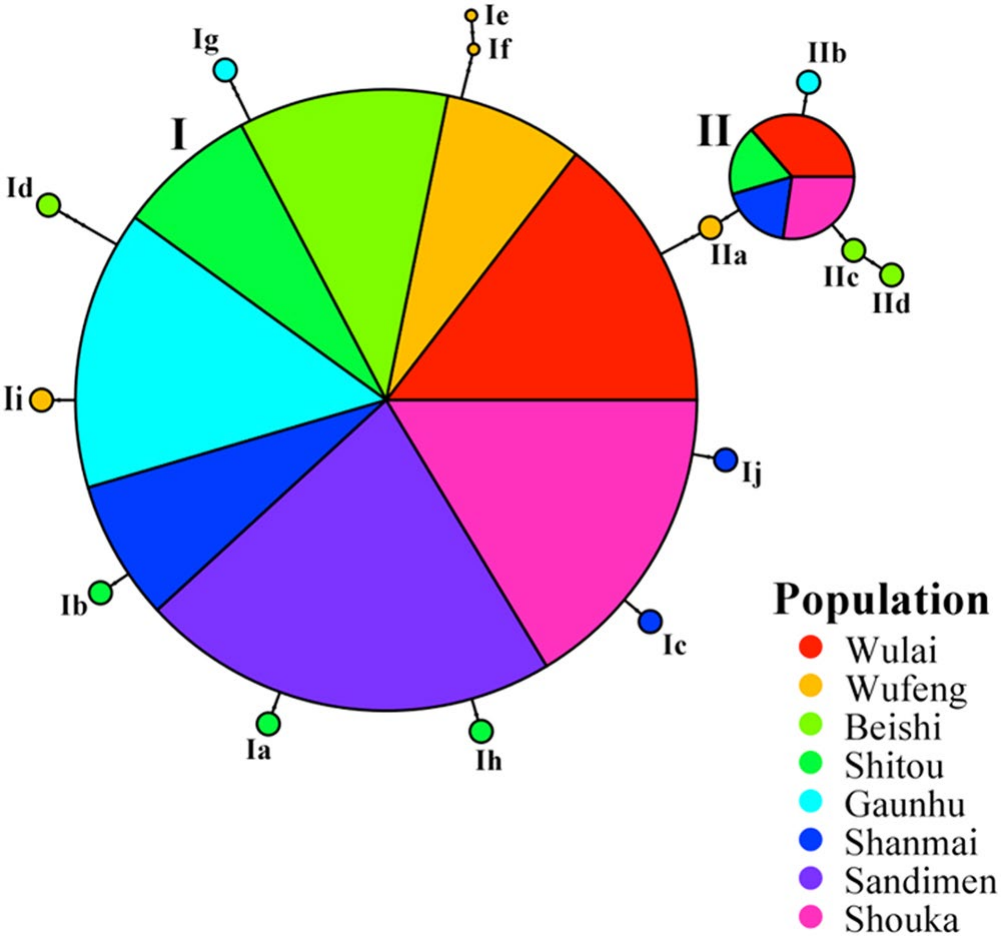
Haplotype network for *Musa basjoo* var. *formosana* based on nuclear superoxide dismutase gene intron 2 sequence data. The size of a circle corresponds to the haplotype frequency. Each line between haplotypes represents a mutational step; the dot represents another mutational step between haplotypes.

We obtained 92 sequences of the second intron of Cu/Zn *SOD2* with an aligned length of 1264 bp. No sign of recombination was found examined within populations. However, one non-significant recombination event (*P* = 0.876) was found between nucleotide sites 15 and 510 when total samples were analyzed. In addition, 2 of 46 samples collected from populations Shouka and Wufeng were heterozygotes and one single-base pair indel was found in the aligned sequences. Sixteen haplotypes were identified in the 92 sequences analyzed (Table 1 and Fig. 2). The number of haplotypes ranged from 1 to 5 for each population. The most common haplotype (haplotype I) was found in all populations examined and the second most common haplotype (haplotype II) was found in four populations (populations Shanmai, Shitou, Shouka, and Wulai) (Table 1 and Fig. 2). Populations Shitou and Wufeng had the greatest number of haplotypes and the population Sandimen had only one haplotype (the most common haplotype I). Although mismatch distribution of the frequency of pairwise differences among haplotypes did not fit tightly with a population expansion model (Kolmogorov-Smirnov test, *P* < 0.001, Supplementary Fig. S1), the haplotype network displayed “star-like” phylogeny in two separate groups indicating population expansion and subdivision (Fig. 2).

**Figure 3:**
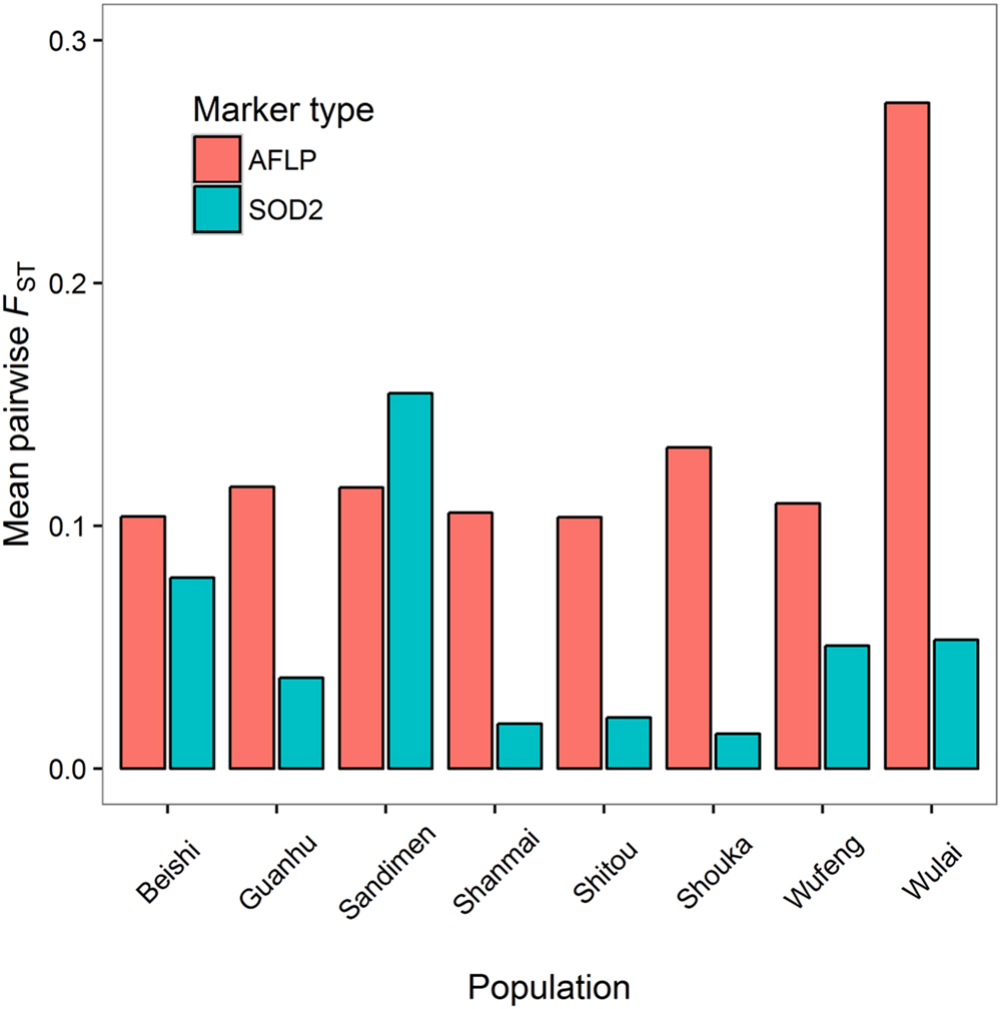
Degree of Mean pairwise *F*_ST_ values of each population in comparison with those of the remaining populations based on the second intron sequences of *Cu/Zn SOD*2 and amplified fragment length polymorphic variation.

Population pairwise *F*_ST_ values calculated from Cu/Zn *SOD2* second intron sequence variation was mostly low (Supplementary Table S1) and averaged 0.053. The southern population Sandimen showed the highest mean pairwise *F*_ST_ (=0.154) against the remaining populations (Fig. 3), which may have been the southern glacial refugium for *M. basjoo* var. *formosana*. Table 1 presents the descriptive statistics of nucleotide diversity of the second intron of Cu/Zn *SOD2* include haplotype diversity (*Hd*) and nucleotide diversity *θ*_S_ and *θ*_π_. *Hd* ranged between 0 (population Sandimen) and 0.848 (population Shitou), *θ*_π_ ranged between 0 (population Sandimen) and 0.00173 (population Beishi), and *θ*_S_ ranged between 0 (population Sandimen) and 0.00168 (population Wufeng). The levels of *Hd*, *θ*_π_, and *θ*_S_ were not correlated with the number of sequences (sample size) via Pearson’s correlation test (*Hd*, *r* = −0.497, *P* = 0.209; *θ*_π_, *r* = −0.383, *P* = 0.349; and *θ*_S_, *r* = −0.469, *P* = 0.240).

Neutrality tests based on Tajima’s *D* ^30^ and Fu’s *F*s^31^ revealed non-significant negative values in many populations and even positive values were observed (Table 2). In contrast, for pooled samples, significant negative values in Tajima’s *D* (=−1.6544, *P* = 0.015) and Fu’s *F*s (=−6.8767, *P* = 0.008) were found. Moreover, non-significant raggedness index (*rg*)^32^ was found for all individual populations and the pooled samples (*rg* = 0.0697, *P* = 0.84) (Table 2). Significant values of the sum of square deviations (SSD) statistic were found for several populations (populations Guanhu, Shitou, Shouka, and Wulai) and *R*_2_ index^33^ was non-significant for all individual populations (Table 2). Nevertheless, non-significant SSD (=01976, *P* = 0.19) and significant *R*_2_ index (=0.0416, *P* = 0.04) were found for pooled samples, indicating spatial range expansions^34^. The mean demographic expansion factor (*τ*)^35^, representing the time since the beginning of an expansion, was 1.6167 (0.30225–8.57544, 95% confidence intervals (CIs)). The time at which the expansion event took place was dated with a mean of 21317 (3985–113072, 95% CIs) years before present for *M. basjoo* var. *formosana*, which corresponded roughly to the time frame at the end of the LGM, from 25,000–18,000 years before the present^2, 5, 36, 37^.

**Table 2:** Neutrality test statistics and mismatch analysis based on the second intron sequences of *Cu/Zn SOD2* and summary of genetic diversity based on 521 amplified fragment length polymorphic loci of eight populations of *Musa basjoo* var. *formosana*.

	Beishi	Guanhu	Shandimen	Shanmai	Shitou	Shouka	Wufeng	Wulai	Total
**Cu/Zn** ***SOD2*** **second intron**									
Tajima’s *D* (*P* value)	0.3743 (0.695)	−0.2986 (0.422)	NA	0.0235 (0.547)	−0.3237 (0.406)	0.7722 (0.779)	−0.2792 (0.397)	1.5227 (0.943)	−1.6544* (0.015)
Fu’s *Fs* (*P* value)	1.1366 (0.742)	1.5988 (0.827)	NA	0.7370 (0.675)	−0.0792 (0.459)	3.6988 (0.957)	−0.2519 (0.411)	4.2310 (0.967)	−6.8767** (0.008)
*rg* (*P* value)	0.2323 (0.12)	0.3315 (0.90)	NA	0.1664 (0.37)	0.1625 (0.44)	0.6839 (0.96)	0.1037 (0.58)	0.7355 (0.94)	0.0697 (0.84)
SSD (*P* value)	0.0785 (0.11)	0.4187** (0.00)	NA	0.0652 (0.27)	0.1819* (0.02)	0.3347** (0.00)	0.0313 (0.54)	0.4702** (0.00)	0.1976 (0.11)
*R*_2_ (*P* value)	0.1818 (0.63)	0.1515 (0.19)	NA	0.1778 (0.44)	0.1515 (0.30)	0.2045 (0.67)	0.1564 (0.22)	0.2424 (0.87)	0.0416* (0.04)
**AFLP**									
*N*	16	15	17	8	15	19	13	18	15.1
*%P*	65.6	52.2	56.8	75.4	64.1	56.0	73.3	50.7	63.1
*N*_*p*_ *(N*_*fp*_ *)*	0 (0)	0 (0)	0 (0)	0 (0)	0 (0)	0 (0)	2 (0)	14 (0)	2 (0)
*HE*	0.280	0.241	0.259	0.324	0.290	0.231	0.336	0.248	0.276
*SE (HE)*	0.0085	0.0082	0.0083	0.0082	0.0079	0.0078	0.0071	0.0072	0.0079

^*^ *P* < 0.05, **P < 0.01 *N*, number of samples; *%P*, percent of polymorphic loci at the 5% level; *H*_E_, Nei’s gene diversity.

### AFLP diversity and differentiation

Twelve primer pairs generated a total of 521 AFLP loci in the entire sample with an overall repeatability of 94.68% (Supplementary Table S2). The proportion of AFLP polymorphic loci ranged from 50.7% (population Wulai) to 75.4% (population Shanmai) with an average value of 63.1% (Table 2). The level of Nei’s genetic diversity (*H*_E_)^38^ averaged 0.276 and ranged from 0.231 (population Shouka) to 0.336 (population Wufeng) (Table 2). The mean *H*_E_ was 0.276.

**Figure 4:**
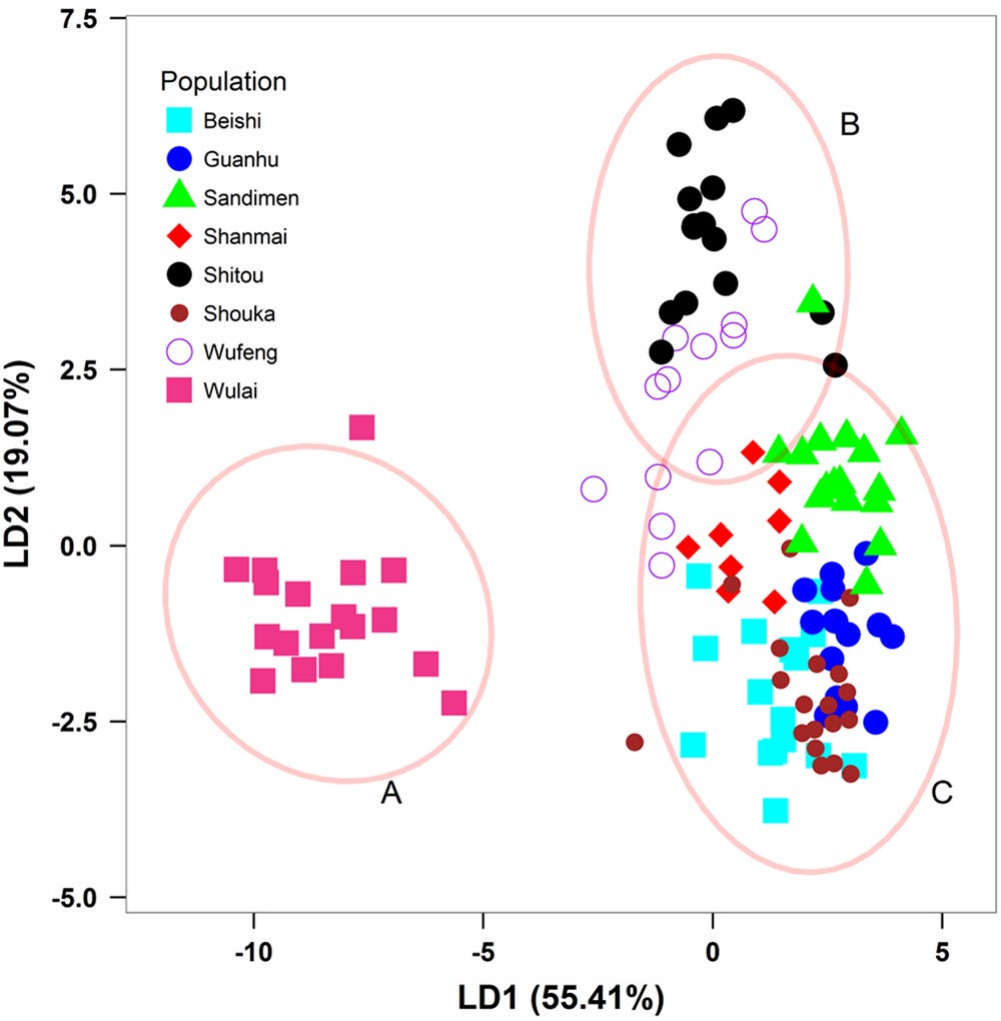
Scatter plot of the first two linear discriminants (LDs) from the discriminant analysis of principal components based on 521 amplified fragment length polymorphic loci for individuals of eight populations of *Musa basjoo var. formosana*. The ellipses represent 95% confidence intervals distinguishing the three genetic clusters A, B, and C.

In AFLP data, the northern population Wulai had the largest average pairwise *F*_ST_ against the remaining populations (mean *F*_ST_ = 0.274, Fig. 2 and Supplementary Table S1). In the HICKORY analysis^39^, the inbreeding coefficient (*f* ) = 0 model, which estimated *θ* ^B^ (an *F*_ST_ analogue) with *f* = 0 best fitted the data by having the lowest deviance information criterion (DIC) and $\overline{{\rm{D}}}$ (a measure of how well the model fits the data) values, but there was little difference in the DIC values between the *f* = 0 model and the next best model (full model) (13771.7 vs. 13772.4) (Supplementary Table S3). The full model estimated an *f* of 0.0789 (0.0091–0.1728, 95% CIs). These results suggested that the degree of inbreeding was low in *M. basjoo* var. *formosana*. The *M. basjoo* var. *formosana* populations were moderately structured according to HICKORY estimates based on the *f* = 0 and full model (*θ* ^B^ = 0.1354 and 0.1429, respectively). The moderate level of genetic differentiation between populations estimated with HICKORY was consistent with the values estimated with AFLP-SURV^40^ (average pairwise *F*_ST_ = 0.1284, Supplementary Table S1) and hierarchical analysis of molecular variance (AMOVA) (*Φ*_ST_ = 0.2117, *P* < 0.001, Supplementary Table S4). When the population Wulai was excluded (because it was highly differentiated from other populations based on DAPC analysis; see result in the following section, Fig. 4), AMOVA revealed a *Φ*_ST_ value of 0.1308 (*P* < 0.001). AMOVA *Φ*_ST_ value based on the three DAPC clusters was 0.2091 (*P* < 0.001).

In the STRUCTURE analysis^41^, the maximal *ΔK* value (change in the log probability) occur at *K* = 2 (Supplementary Fig. S2). However, the highest mean log likelihood (*Ln*P(D)) was obtained when *K* = 8. In the LEA analysis^42^, the minimal cross-entropy was lowest when *K* = 7 (Supplementary Fig. S3). However, analysis based on discriminant analysis of principal components (DAPC)^43^ revealed three genetic clusters with the first two linear discriminants described 74.48% of the total AFLP genetic variation (Fig. 3). DAPC results provided prominent phylogenetic breaks among the clusters. In addition, the values of symmetric similarity coefficient^44^ (SSC) were higher when *K* = 2, 3, and 4 (0.995, 0.986, and 0.983, respectively) and lower when *K* ≥ 5 (Supplementary Fig. S4). Therefore, *K* = 3 could be the most probable genetic clustering scenario according to the results of DAPC, STRUCURE, and LEA analyses (Fig. 3 and Supplementary Fig. S5).

### Effect of environmental variables on AFLP variation among populations

Seven environmental variables, including annual mean temperature (BIO1), annual precipitation (BIO12), mean wind speed (WS_mean_), normalized difference vegetation index (NDVI), soil pH, soil moisture index (TMI)^45^, and wet days (Supplementary Table S5) were retained as explanatory variables for AFLP variation. Variation partitioning^29^ revealed a vast amount of unexplained variation (80.94%, fraction [d]), and the proportion of explained variation was 19.06% (fraction [a+b+c]) (Supplementary Table S6). Within the explained variation, 10.81% (*P* < 0.001) explained by pure environmental variables (fraction [a]) and 8.25% (*P* < 0.001) by geographically structured environmental variables (fraction [b]). No AFLP variation attributed to pure geographical difference (fraction [c]) was found.

### AFLP loci potentially evolved under selection and test for association with environmental variables

**Figure 5:**
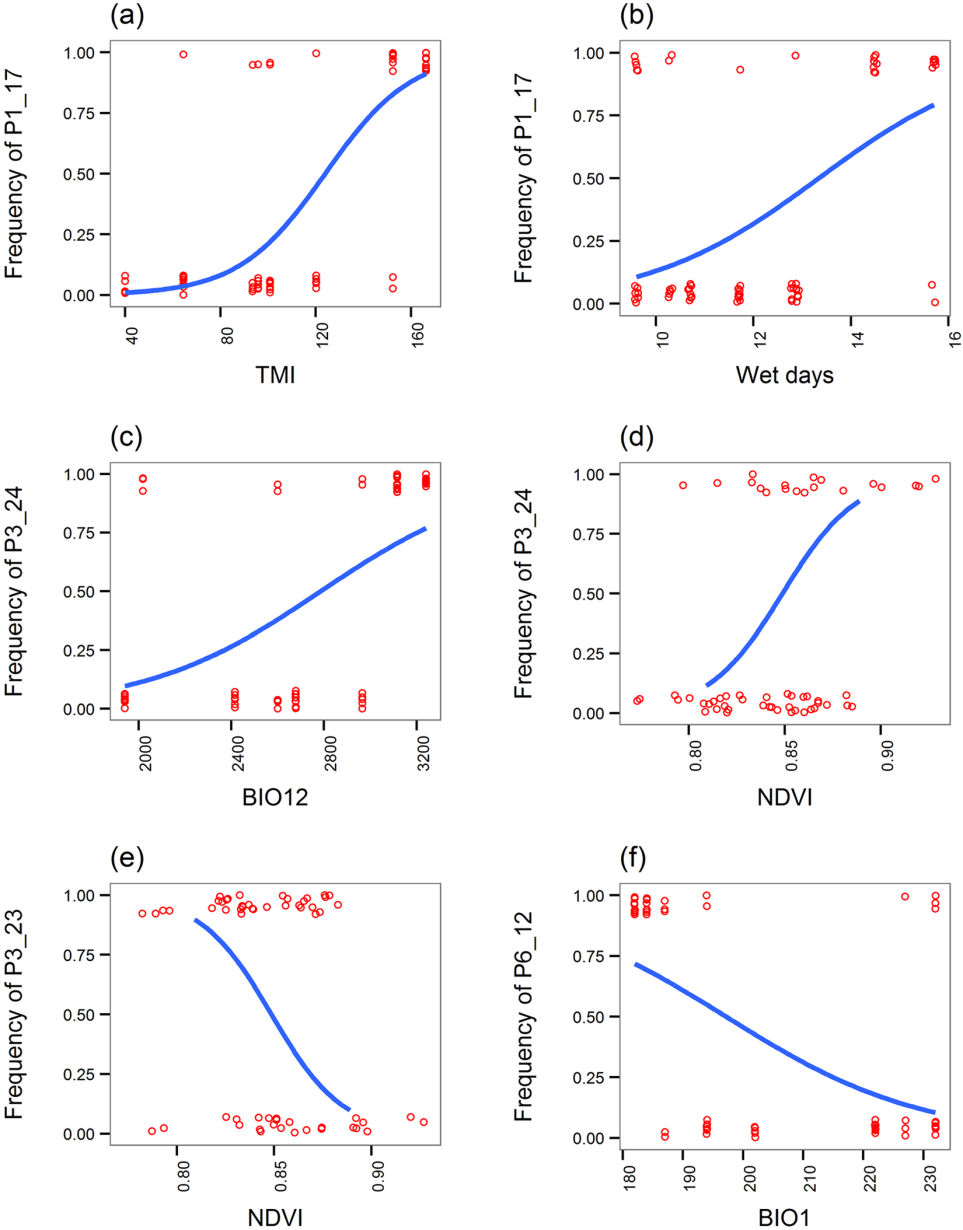
Logistic regression plots of four AFLP loci (P1_17, P3_23 P3_24, and P6_12) potentially evolved under selection against values of specific environmental variables. Logistic regression depicts significant positive relationships of locus P1_17 with environmental variables TMI (**a**) and wet days (**b**) (pseudo-*R* ^2^ = 0.251, *P* < 0.0001; pseudo-*R* ^2^ = 0.106, *P* = 0.0025, respectively) and of locus P3_24 with environmental variables BIO12 (**c**) and NDVI (**d**) (pseudo-*R* ^2^ = 0.094, *P* = 0.00897; pseudo-*R* ^2^ = 0.105, *P* = 0.00897, respectively), and significant negative relationship of locus P3_23 with environmental variable NDVI (**e**) (pseudo-*R* ^2^ = 0.115, *P* = 0.0056) and of locus P6_12 with environmental variable BIO1 (**f**) (pseudo-*R* ^2^ = 0.101, *P* = 0.00368). BIO1, annual mean temperature; BIO12, annual precipitation, NDVI, normalized difference vegetation index; TMI, Thornthwaite moisture index; wet days, number of days with > 0.1 mm of rain per month.

We performed outlier detection for AFLP loci potentially evolved under selection with two neutrality test methods: DFDST and BAYESCAN^46, 47^. Multiple univariate logistic regression was used to test for correlations of frequencies of AFLP loci with values of environmental variables using Samβada^48^. DFDSIT and BAYESCAN identified six (1.15%) and five (0.96%) AFLP loci that potentially evolved under selection (Table 3). Samβada analysis found seven AFLP loci strongly correlated with environmental variables (Table 3). We considered four AFLP loci (P1_17, P3_23, P3_24, and P6_12) as adaptive loci potentially evolved under selection because they were identified by either neutrality test method and correlated strongly with environmental variables. Four AFLP loci that identified as outliers by DFDIST or BAYESCAN and correlated strongly with environmental variables, logistic regression plots were drawn (Fig. 5). In the Samβada analysis, significant positive relationships were found for locus P1_17 with soil moisture and wet days (pseudo-*R* ^2^ = 0.251, *P* < 0.0001; pseudo-*R* ^2^ = 0.106, *P* = 0.0025, respectively) and locus P3_24 with annual mean temperature and NDVI (pseudo-*R* ^2^ = 0.094, *P* = 0.00897; pseudo-*R* ^2^ = 0.105, *P* = 0.00897, respectively). Significant negative relationships were found between locus P3_23 and NDVI (pseudo-*R* ^2^ = 0.115, *P* = 0.0056) and between locus P6_12 and annual mean temperature (pseudo-*R* ^2^ = 0.101, *P* = 0.00368). Samβada analysis reported several types of pseudo-*R* ^2^ values, we adopted the Nagelkerke pseudo-*R* ^2^ because its calculation is based on log likelihoods rather than on residual deviance and scaled approximately from 0 to 1 equivalent to the unadjusted *R* ^2^ in linear regression^49^.

**Table 3:** Amplified fragment length polymorphic (AFLP) loci identified potentially evolved under selection by DFDIST and BAYESCAN neutrality test methods and association of allelic frequencies with values of environmental variables.

Locus	DFDIST^a^	BAYESCAN^b^ (log_10_ (PO))	Samβada^c^ (pseudo-*R* ^2^, *P*-value)
P1_17	X		TMI (0.251, <0.0001); Wet day (0.106, 0.0025)
P1_19			BIO1 (0.00363)
P2_15			TMI (0.266, 0.0021), Wet days (0.326, 0.0024)
P3_23	X	X (4.699)	NDVI (0.115, 0.0056)
P3_24	X	X (4.398)	BIO12 (0.094, 0.0086), NDVI (0.105, 0.0090)
P4_35			Soil pH (0.241, 0.0053)
P6_12	X	X (1.558)	BIO1 (0.101, 0.0037)
P6_17	X	X (1000)	
P6_25		X (1.889)	
P6_30			WSmean (0.362, 0.0064)
P12_29			BIO1 (0.135, 0.0049)
P12_49	X		

^a^For DFDIST, 95% significance level and 5% false discover rates were used.

^b^For BAYESCAN, a log_10_ (probability odds) (log_10_PO) above 1.5 indicated a very strong outlier with a 5% false discovery rate.

^c^For Samβada, values of Nagelkerke’s pseudo-*R* ^b^ are indicated (Nagelkerke 1991). Statistically significant at *P* < 0.01, corresponding to a confidence threshold after Bonferroni corrections of 2.741 x −6.

BIO1, annual mean temperature; BIO12, annual precipitation; NDVI, normalized difference vegetation index; Wet days, number of days with >0.1 mm of rain per month; WS_mean_, mean wind speed; TMI, soil moisture index.

## Discussion

In this study, we surveyed sequence variation in the second intron of Cu/Zn *SOD2* and AFLP variation to investigate whether the postglacial range expansion occurred in *M. basjoo* var. *formosana* and adaptive evolution in association with the environmental gradients of contemporary populations in this species. Sequence variation in the second intron of Cu/Zn *SOD2* suggested range expansion since the LGM. A vast amount of unaccounted variation was observed, which is typical for RDA multivariate analysis^50^. In addition, a considerable amount of unexplained variation could also be attributed to random processes triggered by ecological drift and dispersal, non-spatially structured biological, and/or unmeasured environmental differences^11, 51^. In addition, the natural landscapes of Taiwan have been dramatically altered by humans, particularly in the lowlands, which may have played a role in influencing genetic variation surveyed in *M. basjoo* var. *formosana*. Nevertheless, a significant amount of AFLP variation can be explained by the environmental variables. Local adaptation in *M. basjoo* var. *formosana* populations was suggested by the AFLP loci that were potentially evolved under selection in association with environmental variables, including annual mean temperature, annual precipitation, surface coverage activity (NDVI), soil moisture, and number of days with > 0.1 mm of rain per month (wet days).

Glaciers have had a serious impact on the current distribution of plant species and most subtropical species have retreated toward the tropics or warmer lowland areas^2^. The discrepancy in detecting population expansion for individual populations could be because of either population reduction, population subdivision, a recent bottleneck, or migration^52^. Our data revealed that the mismatch distribution for the frequency of pairwise differences among haplotypes did not fit tightly with a population in the expansion model (Kolmogorov-Smirnov test, *P* < 0.001, Supplementary Fig. S1), which indicates diminishing populations or structured sizes of *M. basjoo* var. *formosana* ^53, 54^. In un-subdivided populations, molecular signature characteristics of sudden expansions might not be observed, even when populations had expanded by several orders of magnitude after the LGM^55^. Although no consistent evidence for population growth was found for individual populations, our results showed evidence for historical spatial range expansion in pooled samples because of significant negative values of neutrality test statistics (Tajima’s *D* = −1.6544, *P* = 0.015; Fu’s *F*s = −6.8767, *P* = 0.008) (Table 2). Moreover, the hypothesis of historical spatial range expansion could not be rejected in pooled samples based on non-significant small *rg* value (*rg* = 0.0697, *P* = 0.84)^32^, non-significant SSD (SSD = 00096, *P* = 0.81)^56^, and significant *R*_2_ (*R*_2_ = 0.0416, *P* = 0.04)^33^ (Table 2).

Based on the average *F*_ST_ value for each population in comparison with the remaining populations, glacial refugia of plant species have been inferred in many plant species in Taiwan. The most divergent populations can be found located in the north-central and south (particularly in the southeastern part) parts of Taiwan for tree species^6^. Populations served as refugia in the north-central part of Taiwan were found for tree species, including *T. aralioides* ^17^, *Cu. koinishii* ^57^, *Ca. carlesii* ^18^, *Machilus thunbergii* ^58^, *Ma. Kusanoi* ^58^, and *Ci. kanehirae* ^6^ based on chloroplast DNA variation; and *Ci. kanehirae* ^19^ and *Quercus glauca* ^59^ based on nuclear DNA variation. Species with southern glacial refugia include *Cy. glauca* ^15^, *T. aralioides* ^17^, *Ca. Carlesii* ^18^, *Ma. thunbergii* ^58^, *Ma. Kusanoi* ^58^, and *Ci. kanehirae* ^6^ based on chloroplast DNA variation; and *Ci. kanehirae* ^19^ and *Q. glauca* ^59^ based on nuclear DNA variation. Our results in *M. basjoo* var. *formosana* reflect recolonization events after the latest glacial period that probably originated from the southern population Sandimen due to its highest mean pairwise *F*_ST_ (=0.154) against the remaining populations based on sequence variation of the second intron of Cu/Zn *SOD2* (Fig. 3). However, the population Wulai had the highest average *F*_ST_ based on AFLP variation (Fig. 3) and clearly distinguished from other populations according to the DAPC genetic clustering analysis (Fig. 4), suggesting that the population Wulai may have been the northern glacial refugium for *M. basjoo* var. *formosana*.

In comparison with other angiosperm species that occurred in Taiwan, *M. basjoo* var. *formosana* had lower nucleotide diversity in the Cu/Zn *SOD2* second intron (*Hd* = 0.628, *θ*_π_ = 0.00119, and *θ*_S_ = 0.00280) compared to introns of chalcone synthase (*Chs*) and leafy (*Lfy*) genes in *Ci. kanehirae* (*Chs*: *Hd* = 0.841, *θ*_π_ = 0.00716, and *θ*_S_ = 0.00371; *Lfy*: *Hd* = 0.895, *θ*_π_ = 0.00479, and *θ*_S_ = 0.00805)^19^ and intron of glyceraldehyde-3-phosphate dehydrogenase gene in *Q. glacuca* (*Hd* = 0.840 and *θ*_π_ = 0.00500)^59^. However, these comparisons may not be appropriate based on only one gene and the gene sequences compared between species were different. Nevertheless, the level of sequence nucleotide diversity reflects the past demographic events in natural populations. Although populations Sandimen and Wulai may have been the glacial refugia for *M. basjoo* var. *formosana*, populations Beishi, Shitou, and Wufeng located in north and central Taiwan across the CMR had higher levels of nucleotide diversity (Table 1). These results suggested that populations Beishi, Shitou, and Wufeng may be the melting pots of diversity^5^. However, the migration routes may have occurred mostly northward from the southern refugium (population Sandimen) and between adjacent populations except for the population Wulai; and scarcely southward from the northern refugium (population Wulai). This is because the population Wulai had a high average *F*_ST_ (=0.274) in comparison with all other populations based on AFLP variation (Supplementary Table S1) and individuals of the population Wulai are clearly differentiated from individuals of all other populations (Fig. 4). Compared to other populations, the population Wulai is identified as potential refugium located in higher elevation because the rugged geographic topology might serve as an insular area for the survival of this population during the LGM.

AFLP displayed higher level of genetic diversity (average *H*_E_ = 0.276) in *M. basjoo* var. *formosana* compared with other broadleaf tree species occurred in Taiwan, such as *Rhododendron oldhamii* (average *H*_E_ = 0.216)^11^ and species in the genus *Salix* (average *H*_E_ = 0.166)^12^. *M. basjoo* var. *formosana* also had a relatively high level of AFLP variation compared to the average *H*_E_ (=0.230) for 13 plant species summarized in Nybom^60^. Moreover, the level of AFLP variation of *M. basjoo* var. *formosana* was comparable to that of *M. balbisiana*, another wild banana species, occurred in China (average *H*_E_ = 0.241)^61^. Patterns of genetic variation in contemporary populations of a species are influenced by the historical processes that shaped the distribution of a species^1, 2^, by the landscape ecological properties^11, 62^, and by life history traits^63^. High levels of *H*_E_ in *M. basjoo* var. *formosana* may have been related to the low degree of inbreeding revealed by the HICKORY analysis (Supplementary Table S3). Long life span and predominant outcrossing by animal pollinators may account for high AFLP diversity in wild bananas^61, 64^. The potential build-up of genetic variation during the course of expansion since the LGM, particularly under climate change conditions, is important for species’ survival facing global climate change^27^. *M. basjoo* var. *formosana* harbors a substantial amount of AFLP variation and is an important resource for populations to adapt to changing environmental conditions under natural selection.

In *M. basjoo* var. *formosana*, pure environmental and spatially structured environmental factors explained a significant amount of AFLP variation (Supplementary Table S6), which suggests that environments play important roles in influencing genetic variation of this species. Environmental variables such as temperature, precipitation, surface coverage activity, soil moisture, and wet days are the most important ecological drivers influencing genetic variation of *M. basjoo* var. *formosana* and correlated strongly with four AFLP loci based on multiple univariate logistic regression analysis (Table 3 and Fig. 5), indicating fitness-related change in AFLP variation. Temperature and precipitation are commonly found to be the ecological drivers strongly correlated with adaptive AFLP variation for many plant species that occur in Taiwan, such as *R. oldhamii* ^11^, *Keteleeria davidiana* var. *formosana* ^9^, and *Salix* species^12^. Temperature and precipitation appeared to be common ecological drivers for adaptive AFLP variation in natural populations of various plant species^8, 65–68^.

Soil properties can explain a non-negligible proportion of the spatial distribution of tree species^69^ and influence genetic variation among populations of tree species^70–72^. Soil moisture is associated with allozyme genotypes at the glycerate dehydrogenase locus and may play an important role in the adaptation of *Pinus edulis* ^70^; and with AFLP loci such as in *Fagus sylvatica* ^71^ and *Eperua falcate* ^72^. NDVI is a measure of surface coverage activity indicating the level of vegetation greenness and acts as a proxy for a biotic competitive environment. NDVI, estimated with the moderate resolution imaging spectroradiometer, has been shown to be linear with the fraction of absorbed photosynthetically active radiation (fPAR)^73^, which was not retained as explanatory variable in this study (Supplementary Methods). NDVI and/or fPAR can be influential factors acting on the genetic variation of a species in response to interactions with other species in a local ecological community^74^; and has been shown to be correlated with population adaptive divergence^12^ and adaptive divergence between species^12, 13^.

Understanding environmental variables acting as ecological drivers and playing roles in shaping the contemporary gene pool structure of *M. basjoo* var. *formosana* from experienced postglacial range expansion is important. Our results suggest that there are two glacial refugia located in the northern and southern part of the *M. basjoo* var. *formosana* distribution range. Postglacial expansion confronting ecological discontinuity may have triggered the evolution of environmentally associated AFLP variation underlying local adaptation. Although a vast amount of AFLP variation was attributed to residual effects, a significant amount of explained variation attributed to environmental effects was found. In conclusion, environmental variables include temperature, precipitation, soil moisture, surface coverage activity, and wet days may have been the most important ecological drivers for adaptive evolution of postglacial expanded *M. basjoo* var. *formosana* populations, and have played roles in shaping the current distributions of this species.

## Methods

### Sampling

A total of 112 individuals from eight populations of *M. basjoo* var. *formosana* were collected (Fig. 1; Tables 1 and 2). All samples were subjected to AFLP genotyping and 46 were used to obtain the Cu/Zn *SOD2* second intron sequences.

### Cloning and sequencing of Cu/Zn *SOD2* second intron

The extraction of genomic DNA and total RNA from leaves followed the methods of Dellaporta *et al*.^75^ and Clendennen and May^76^, respectively. First-stranded cDNA was synthesized using total RNA and reverse transcribed (MMLV reverse transcriptase, Promega), and amplified using RACE kit (Invitrogen) with degenerate primers (SOD-F1, 5′-CTCRMKCCDGGNCTCCATGGCTTCC-3′; and SOD-R1, 5′-TTTCCKTCRTCRCCMRCATG-3′). RACE products were then ligated into pGEM-T vector (Promega) and transformed to *E*. *coli*. Two full-length Cu/Zn *SOD* coding sequences were cloned and sequenced (mbCSD1: ABI34606 and mbCSD2: ACY24898).

The *Bam*HI-, *Bgl*II-, *Eco*RI-, or *Sac*I-digested genomic DNA was self-religated and used for the first inverse polymerase chain reaction (IPCR) with CSD-F2 and CSD-R2 primers (5′-GGTGATACCACCAACGGCTGC-3′ and 5′-GCAGCCGTTGGTGGTATCACC-3′). The first IPCR was performed at 94 °C for 6 min, 25 cycles of 20 sec at 94 °C, 15 sec at 60 °C, 2 min at 72 °C, followed by 5 min at 72 °C. The first IPCR amplified fragments were used as template in the second IPCR with CSD-F3 (5′-CACGTGATGAGGAACGACATGC-3′) and CSD-R3 (5′-GCAGCCGTTGGTGGTATCACC-3′) primers in a PCR reaction at 94 °C for 4 min, 25 cycles of 30 sec at 94 °C, 30 sec at 60 °C, 2 min at 72 °C, followed by 5 min at 72 °C. The second IPCR products were subcloned to pUC119 and sequenced (Genbank accession numbers for genomic sequences of Cu/Zn *SOD1* and Cu/Zn *SOD2* are DQ866814 and GU045759, respectively).

An initial screening found no variation in introns of Cu/Zn *SOD1* and Cu/Zn *SOD2* except the second intron of Cu/Zn *SOD2* in several samples from different populations. Specific CSD2-F1 (5′-CTCCACTGGTAAACCCTCG-3′) and CSD2-R1 (5′-GAGGTCCTGCATGACAACAAG-3′) primers were used to amplify Cu/Zn *SOD2* second intron sequences of 46 individuals in PCR reactions with 6 min at 94 °C for 6 min, followed by 30 cycles of 30 sec at 94 °C, 30 sec at 57 °C, 1.5 min at 72 °C, and 5 min holding at 72 °C. The sequential PCR fragments were ligated into pGEM-T vector, transformed into *E. coli*, and double sequenced using M13 primers and haplotype sequences deposited (GenBank accessions: KX688826~KX688841).

### Sequence alignment, nucleotide diversity, haplotype, and demography

Sequences were aligned with Clustal X^77^ and nucleotide diversity *θ*_π_ and *θ*_S_, haplotype diversity (*Hd*), *R*_2_ index, and pairwise mismatch distribution estimated using DnaSP v5.0^78^. *θ*_π_ and *θ*_S_ were calculated based on the average pairwise number of differences between sequences and the number of segregating sites per sequence, respectively. Population pairwise *F*_ST_, raggedness (*rg*) index of observed mismatch distribution, and neutrality statistics (Tajima’s *D* and Fu’s *F*s) were estimated using Arlequin v3.5^79^. Moreover, goodness-of-fit of the observed mismatch distribution to that expected under the spatial expansion model was tested using the sum of square deviations (SSD) statistic with Arlequin. We generated a haplotype network with the R “pegas” package^80, 81^.

Neutrality statistics are nearly zero in constant-size populations, whereas a significant negative value and significant positive value reflect processes such as population subdivision or recent bottlenecks. Positive *R*_2_ value and significance of coalescent simulation against neutral model indicate population expansion. The raggedness index is a measure that quantifies the smoothness of the observed mismatch distribution and small *rg* values represent a population that has experienced sudden expansion^32^. A significant SSD value indicates departure from spatial expansion^34^.

To estimate the time since the beginning of an expansion, we used *t* = *τ*/2*μk*, where *t* is the time elapsed between initial and current population sizes, *τ* is the estimated number of generations since the expansion, *μ* is the mutation rate, and *k* is the sequence length. A mutation rate of 1.5% per 10^6^ year per site was used^19^. Demographic expansion factor (*τ*) was estimated using Arlequin. We assumed a mean generation time of 2 years for *M. basjoo* var. *formosana*.

### AFLP

Genomic DNA (200 ng) was digested with 5 U *Eco*RI and 5 U *Mse*I (Yeastern Biotech, Taipei, Taiwan) for AFLP genotyping^28^. Digested products were ligated with *Eco*RI (0.5 μM) and *Mse*I (5 μM) adaptors in a 10-μl ligation reaction mixture using 30 U T4 DNA ligase (Yeastern Biotech) at 22 °C for 1 hr. Preselective amplification was performed in a total volume of 10-μl reaction buffer, including 1 μl of the digested sample (1:9 dilution), 100 nM of *Eco*RI (E00: 5′-GACTGCGTACCAATTC-3′) and *Mse*I (M00: 5′-GATGAGTCCTGAGTAA-3′) primers (Supplementary Table S2), 0.25 mM dNTPs, and 1 U *Taq* (Bernardo Scientific, Taipei, Taiwan). The thermal cycling parameters for preselective amplification were as follows: 2 min at 72 °C and 3 min at 94 °C, followed by 25 cycles of 30 sec at 94 °C, 30 sec at 56 °C, and 5 min holding at 72 °C. Twelve *Eco*RI (labeled with FAM) and *Mse*I selective primer combinations (E00 and M00 primers with three additional bases; Supplementary Table S2) were used in selective amplification. Selective amplification was performed with 1 μl diluted preselective amplification product (1:9 dilution) in a 10-μl 1 x PCR buffer containing *Eco*RI and *Mse*I (both 100 nM) selective primers, 0.25 mM dNTPs, 0.75 U *Taq* with an initial holding at 94 °C for 3 min, followed by 12 cycles of 30 sec at 94 °C, 30 sec at 65 °C with a 0.7 °C touchdown per cycle and 1 min at 72 °C, followed by 24 cycles of 30 sec at 94 °C, 30 sec at 56 °C, 1 min at 72 °C, with a final 5 min holding at 72 °C. Selective amplification were visualized on an ABI PRISM 3100 sequencer (Applied Biosystems, Foster City, CA, USA). AFLP fragments were scored with Peak Scanner v2.0 (Applied Biosystems) for each individual in the range of 150–500 bp with relative fluorescent unit threshold set at 100, and genotyping error rate estimated (Supplementary Table S2). AFLP genotyping data are available in the Supplementary Data.

### Environmental variables

Seven environmental variables, including annual mean temperature (BIO1), annual precipitation (BIO12), number of days with > 0.1 mm of rain per month (wet days), mean wind speed (WS_mean_), normalized difference vegetation index (NDVI), soil pH, and soil moisture index (TMI) were retained as explanatory variables (Supplementary Methods and Supplementary Table S4).

### AFLP diversity, structure, and relationships

Nei’s genetic diversity (*H*_E_)^38^ and pairwise *F*_ST_ based on a Bayesian method with non-uniform prior distribution of allele frequencies^82^ was calculated using AFLP-SURV^40^. The numbers of private and fixed private bands were calculated using FAMD^83^. Hierarchical analysis of molecular variance (AMOVA) was performed with the “poppr” package of R^84^ and significance tested based on 9999 permutations with the “ade4” package of R^85^. The Bayesian program, HICKORY v1.1^39^, was used to estimate an *F*_ST_ analogue (designated *θ* ^B^) from dominant markers accounting for inbreeding coefficient (*f* ) using default settings for sampling and chain length parameters (burnin = 5,000; samples = 100,000; thinning = 20). Four models, including a full model, *f* = 0 model, *θ* ^B^ = 0 model, and *f*-free model were fitted with the AFLP data (Supplementary Methods).

Initial detection of genetic structure of *M. basjoo* var. *formosana* was carried out with two assignment test methods: Bayesian clustering^41^ and sparse non-negative factorization (sNMF)^42^ methods. Bayesian clustering method adapted for dominant markers implemented in STRUCTURE^41^ was used to estimate an individual’s probability of belonging to a homogeneous cluster (K populations). An admixture model was adopted and tested with ten runs, for *K* ranging from 1 to 9, with 10^6^ iterations and 10^5^ burn-in steps. R package “pophelper”^86^ was used to summarize mean log likelihood (*Ln*P(D)), change in the log probability (*ΔK*)^87^, and symmetric similarity coefficient (SSC)^88^ to evaluate the fit of different clustering scenarios. Genetic assignment of individuals was also inferred for *K* values ranging from 1 to 9 based on sNMF algorithm and least-squares optimization with the “LEA” package of R^42^. The settings for the LEA analysis were: regularization parameter = 100, iterations = 200, and repetitions = 10 with other arguments set to defaults, and the best *K* evaluated with the means of minimal cross-entropy.

The relationships between individuals and populations of *M*. *basjoo* var. *formosana* were also visualized based on the discriminant analysis of principal components (DAPC) using the R “adegenet” package^43, 89^. DAPC first performed a principal component analysis (PCA), and an optimal number of PCs was estimated and retained for further discriminant analysis.

### Effect of environmental variables on AFLP variation

Redundancy analysis (RDA) was used to partition AFLP variation and explained by environmental and geographical variables using the R “vegan” package^90^ and significance tested with 9,999 permutations. Proportions of AFLP genetic variation (adjusted *R* ^2^) explained by pure environmental variables, geographically structured environmental variables, pure geographical variables, and residual components were estimated^29^. The longitude and latitude of sample localities were used as geographical variables.

### AFLP outlier detection and association with environmental variables

Two *F*_ST_ outlier detection methods (DFDIST and BAYESCAN) were used to identify AFLP loci that potentially evolved under selection. DFDIST implemented in Mcheza software^46^ estimated allele frequencies based on the Bayesian approach^82^ and the highest and lowest 30% of the initial *F*_ST_ were removed for calculating the mean *F*_ST_. Outliers were identified by observed *F*_ST_ and *H*_E_ compared to simulated neutral distributions generated using 10^5^ iterations of coalescent simulations. Loci with unusually high *F*_ST_ values at the 95% confidence level by applying a false discovery rate of 5% were considered potentially evolved under selection. BAYESCAN uses a Bayesian likelihood approach via a reversible-jump Markov Chain Monte Carlo algorithm in comparing the selection versus neutrality model to identify AFLP loci that are potentially evolved under selection^47^. Posterior odds (PO), the ratio of posterior probabilities of selection over neutrality, was estimated with the settings of a sample size of 50,000 and thinning interval of 20 among 10^6^ iterations, following 100 pilot runs of 50,000 iterations. When an AFLP locus with log_10_(PO) > 1.5^91^ was considered to have strong evidence for selection.

Samβada^48^ was further used to evaluate the associations between frequencies of AFLP loci and values of environmental variables using logistic regression model; and significant fit was identified based on the Wald and likelihood ratio tests with Bonferroni correction at *P* < 0.01. Given the relationships between values of environmental variables and frequencies of AFLP outlier loci, logistic regression plots were depicted.

## Electronic supplementary material

SUPPLEMENTARY INFORMATION
