# Supplementary material for: Postglacial range expansion and the role of ecological factors in driving adaptive evolution of Musa basjoo var. formosana
Source: Sci Rep. 2017 Jul 13;7:5341. doi: 10.1038/s41598-017-05256-6 (PMC5509723; doi:10.1038/s41598-017-05256-6)
Supplement: Supplementary file 1 — SUPPLEMENTARY INFORMATION [file 41598_2017_5256_MOESM1_ESM.pdf]

SUPPLEMENTARY INFORMATION

"Postglacial range expansion and the role of ecological factors in driving adaptive evolution of *Musa basjoo* var. *formosana*"

Jui-Hung Chen[1,†], Chun-Lin Huang[2,†], Yu-Long Lai[1], Chung-Te Chang[3], Pei-Chun Liao[1], Shih-Ying Hwang[1,*] and Chih-Wen Sun[1,*]

[1] Department of Life Science, National Taiwan Normal University, 88 Tingchow Road, Section 4, Taipei 11677, Taiwan

[2] Laboratory of Molecular Phylogenetics, Department of Biology, National Museum of Natural Science, 1 Guanchien Road, Taichung 40453, Taiwan

[3] Department of Geography, National Taiwan University, 1 Roosevelt Road, Section 4, Taipei 10617, Taiwan

† Contributed equally to this work.

* Corresponding authors: hsy9347@ntnu.edu.tw and cwsun@ntnu.edu.tw

Department of Life Science, National Taiwan Normal University, 88 Tingchow Road, Section 4, Taipei 11677, Taiwan

Tel: +886-2-77346262

Fax: +886-2-29312904

Supplementary Methods:

Environmental variables. Twenty-four climatic variables obtained included five: relative humidity, cloud cover, time of sunshine, wet days (number of days with > 0.1 mm of rain per month), and mean wind speed (recorded in 390 meteorological stations in 1990-2013)

obtained from the Data Bank for Atmospheric Research (DBAR, https://dbar.ttfri.narl.org.tw/) and 19 obtained from the WorldClim database averaged for the 1950-2000 period at 30-sec spatial resolution (~ 1 km) (http://www.worldclim.org/download/)[1]. Monthly mean values for each sample locality of the five climatic variables obtained from the DBAR were estimated by interpolation of nearby meteorological observations at spatial resolution of 1 km using a universal spherical model of the Kriging method in ArcGIS[2]. Thornthwaite moisture index (TMI)[3] is an indicator of water supply representing moisture limitation in an area. This index was calculated based on annual precipitation and annual potential evapotranspiration (derived from annual mean temperature). Soil pH values of sample localities were acquired from data collected in an island-wide soil investigation (n = 1150) between 1969 and 1986[4].

Digital terrain model with a 40 m resolution was used to collect topographic variables, including elevation, aspect, and slope, of sample localities. Estimates of Normalized difference vegetation index (NDVI), enhanced vegetation index (EVI), fraction of absorbed photosynthetically active radiation (fPAR), and leaf area index (LAI) were obtained using the moderate resolution imaging spectroradiometer (MODIS) based on 8-d Terra surface reflectance products for the period 2001-2013 from the Land Process Distributed Active Archive Center (http://lpdaac.usgs.gov). Using a maximum value composite procedure, 156 monthly MODIS images were generated based on 580 images (3-4 images/month). Monthly mean values of each sampling sites were extracted and computed from the environmental layers and MODIS images using ArcGIS. Variance inflation factor (VIF) represents multicollinearity of environmental variables was calculated using the "vegan" package of R[5,6]. Highly correlated environmental variables ($|r| > 0.8$) and any variables with VIFs > 20[7] were removed. Seven environmental variables (annual mean temperature, annual precipitation, wet days, WS_{mean}, NDVI, soil pH, and TMI) were retained as explanatory variables

(Supplementary Table S2).

HICKORY analysis. The Bayesian program, HICKORY8, was used to estimate an F_{ST} analog (designated θ^B) from dominant markers, whereas accounting for the uncertainty associate with the inbreeding coefficient (*f*). Four models were fitted to the AFLP data: (i) both *f* and θ^B are unknown and ≥ 0 (full model); (ii) θ^B is unknown but assumes no inbreeding occurs ($f = 0$ model); (iii) there is no differentiation between populations but f is unknown ($\theta^B = 0$ model); and (iv) inbreeding coefficient *f* is selected at random from a prior distribution and calculates θ^B separately during a Monte Carlo Markov chain (MCMC) run (*f*-free model). All HICKORY analyses were preformed using default settings for sampling and chain length parameters (burnin = 5,000, samples = 100,000, thinning = 20). Pairwise F_{ST} representing interpopulation genetic differentiation between every pair of populations was also estimated with the non-uniform prior distribution Bayesian estimator in AFLP-SURV assuming Hardy-Weinberg equilibrium with $f = 0$ (the best model selected by HICKORY), and significance evaluated with 999 permutations.

Supplemental References:

1. Hijmans, R. J., Cameron, S. E., Parra, J. L., Jones, P. G. & Jarvis, A. Very high resolution interpolated climate surfaces for global land areas. *Int. J. Climatol.* **25,** 1965-1978 (2005).
2. Chang, C. T., Wang, S. F., Vadeboncoeur, M. A. & Lin, T. C. Relating vegetation dynamics to temperature and precipitation at monthly and annual timescales in Taiwan using MODIS vegetation indices. *Inter. J. Remote Sens.* **35,** 598-620 (2014).
3. Thornthwaite, C. W. An approach toward a rational classification of climate. *Geogr. Rev.* **38,** 55-94 (1948).

4. Chang, C. T., Lin, T. C. & Lin, N. H. Estimating the critical load and the environmental and economic impact of acid deposition in Taiwan. *J. Geogr. Sci.* **56,** 39-58 (2009) (In Chinese).
5. Oksanen, J. *et al.* Vegan: community ecology package. R package version 2.0-1. https://cran.r-project.org/web/packages/vegan/ (2011).
6. R Development Core Team. R: A Language and Environment for Statistical Computing, Version 3.0.0. http://www.R-project.org/ (2013).
7. Borcard, D., Gillet, F. & Legendre, P. *Numerical ecology with R* (Springer, New York, 2011).
8. Holsinger, K.E. & Lewis, P. O. Hickory: a package for analysis of population genetic data v1.0. http://www.academia.edu/1839794/HICKORY (2003).
9. Jakobsson, M. & Rosenberg, N. A. CLUMPP: a cluster matching and permutation program for dealing with label switching and multimodality in analysis of population structure. *Bioinformatics* **23,** 1801-1806 (2007).

Supplementary Table S1. Population Pairwise F_{ST} based on sequence variation of the second intron of Cu/Zn *SOD*2 (upper triangle) and 521 amplified fragment length polymorphic loci (lower triangle).

	Beishi	Guanhu	Sandimen	Shanmai	Shitou	Shouka	Wufeng	Wulai
Beishi		0.0494	0.1818	0.0479	0.0554	0.0455	0.0977	0.0727
Guanhu	0.0566		0.0909	0.0080	0.0083	0.0048	0.0422	0.0579
Sandimen	0.0989	0.0769		0.1367	0.0909	0.1818	0.1277	0.2727
Shanmai	0.0232	0.0711	0.0759		0.0000	0.0000	0.0118	0.0000
Shitou	0.0823	0.0646	0.0650	0.0415		0.0000	0.0183	0.0123
Shouka	0.1286	0.0878	0.0772	0.1155	0.1104		0.0108	0.0000
Wufeng	0.0593	0.0881	0.0928	0.0545	0.0711	0.1330		0.0453
Wulai	0.2774	0.3078	0.3069	0.2738	0.2689	0.2694	0.2157	

Supplementary Table S2. Primer combinations and sequences of the three bases additional to the *Eco*RI (5'GACTGCGTACCAATTC3') or *Mse*I (5'GATGAGTCCTGAGTAA3') adaptor used for the AFLP analysis.

Primer	*Eco*RI	*Mse*I	Number of markers	Error rate
P1	AGC	ACT	57	5.32%
P2	AGC	TGT	28	5.63%
P3	AGC	GCA	39	5.31%
P4	AGC	GCG	42	2.88%
P5	AGC	GGC	31	6.25%
P6	AGC	TCC	33	6.19%
P7	AAC	ACT	53	4.28%
P8	AAC	TGT	49	7.17%
P9	AAC	GCA	47	4.85%
P10	AAC	GCG	49	4.38%
P11	AAC	GGC	43	5.61%
P12	AAC	TCC	50	5.93%
Total			521	
Average			43.42	5.32%

The genotyping error rate per locus was calculated from 16 individuals as the ratio of mismatches in 48 replicates to the total number of replicated markers.

Supplementary Table S3. HICKORY analysis of inbreeding and genetic differentiation based on 521 amplified fragment length polymorphic loci of eight populations of *Musa basjoo* var. *formosana.*

	Parameter					
Model	$\bar{D}$	$\hat{D}$	pD	DIC	f (95% CI)	θ^B (SD)
Full	11377.6	8982.92	2394.72	13772.4	0.0789113 (0.0091245-0.172845)	0.142949 (0.006)
$f = 0$	11350.5	8929.36	2421.16	13771.7		0.135373 (0.004)
$\theta = 0$	21339.6	20849.6	490.037	21829.7	0.937472 (0.796355-0.998007)	
f free	11691.7	9064.73	2626.94	14318.6	0.504402 (0.0278119-0.976414)	0.193505 (0.012)

θ^B, is the best Bayesian inference estimate of the proportion of genetic diversity due to differences among populations, and is an analogue to F_{ST}.

$\bar{D}$, is a measure of how well the model fits the data (smaller values indicate a better fit).

$\hat{D}$, is a measure of how well the best point estimate fits the data.

pD, is a measure of model complexity, i.e., the effective number of parameters being estimated (pD = $\bar{D}$-$\hat{D}$).

DIC, deviance information criterion.

f, an estimate of F_{IS}, inbreeding within a population

Supplementary Table S4. Summary of the analysis of molecular variance (AMOVA) based on 521 amplified fragment length polymorphic loci of *Musa basjoo* var. *formosana.*

Source of variation	df	Sum of squares	Percent of variation	*Φ* Statistics	*P* value
Among 8 populations					
Between populations	7	1989.365	14.15681	$\Phi_{ST} = 0.2117$	< 0.001
Within populations	113	6376.734	56.43128		
Total	120	8366.099	71.58809		
Among 3 DAPC clusters					
Between populations	2	1127.730	16.21474	$\Phi_{ST} = 0.2091$	< 0.001
Within populations	118	7238.369	61.34211		
Total	120	8366.099	77.55685		
Without Wulai population					
Between populations	6	1112.167	8.726577	$\Phi_{ST} = 0.1308$	< 0.001
Within populations	92	5567.290	57.992602		
Total	106	6679.456	66.719179		

Supplementary Table S5. Site environmental variables including site names, annual mean temperature (BIO1), Annual precipitation (BIO12), Wet days, mean wind speed (WS_{mean}), normalized difference vegetation index (NDVI), Soil pH, and Thornthwaite moisture index (TMI) of eight populations of *Musa basjoo* var. *formosana.*

Population	BIO1	BIO12	Wet days	WS_{mean}	NDVI	Soil pH	TMI
Beishi	194	2678	15.7	2.5	0.849	5.00	152.4
Guanhu	202	1941	12.9	3.0	0.838	5.00	93.5
Sandimen	222	2966	9.6	2.6	0.843	4.70	120.2
Shanmai	227	2019	12.8	3.2	0.812	5.80	39.9
Shitou	187	2416	11.7	2.3	0.809	4.65	100.8
Shouka	232	3116	10.7	2.6	0.889	4.80	64.4
Wufeng	184	2600	10.3	2.6	0.832	4.10	95.8
Wulai	182	3241	14.5	2.2	0.861	4.80	166.3

Supplementary Table S6. The proportion of genetic variation accounted for non-spatially structured environmental variables [a], shared (geographically-structured) environmental variables [b], pure geographic factors [c] and undetermined component [d].

	Adjusted R^2	F	P
Environmental [a]	0.10806	4.1506	0.001
Environmental + Spatial [b]	0.08251	-	-
Spatial [c]	0	-	-
Residuals [d]	0.80943	-	-
[a + b + c]	0.19057	5.0361	0.001

Proportions of explained variation were obtained from variation partitioning by redundancy analysis (RDA). *F*- and *P*-values are specified wherever applicable.

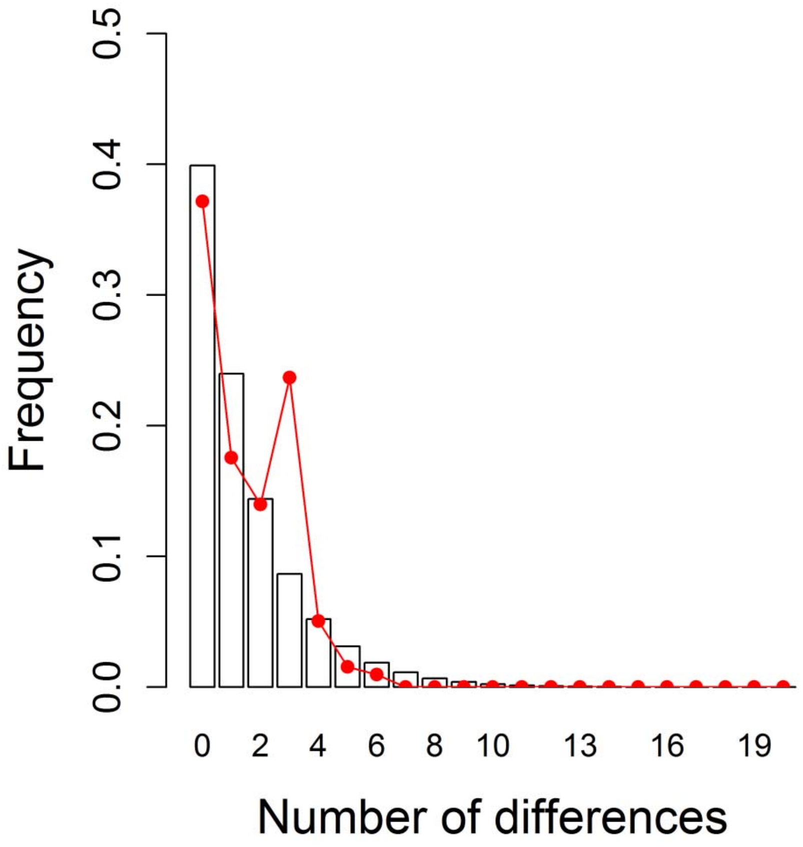

Supplementary Fig. S1. Observed and expected mismatch distributions showing the frequencies of pairwise differences based on nuclear superoxide dismutase gene intron 2 sequence data. The observed distribution (red dots and solid lines) is compared for their goodness-of-fit to a Poisson distribution under a model of sudden expansion (bars).

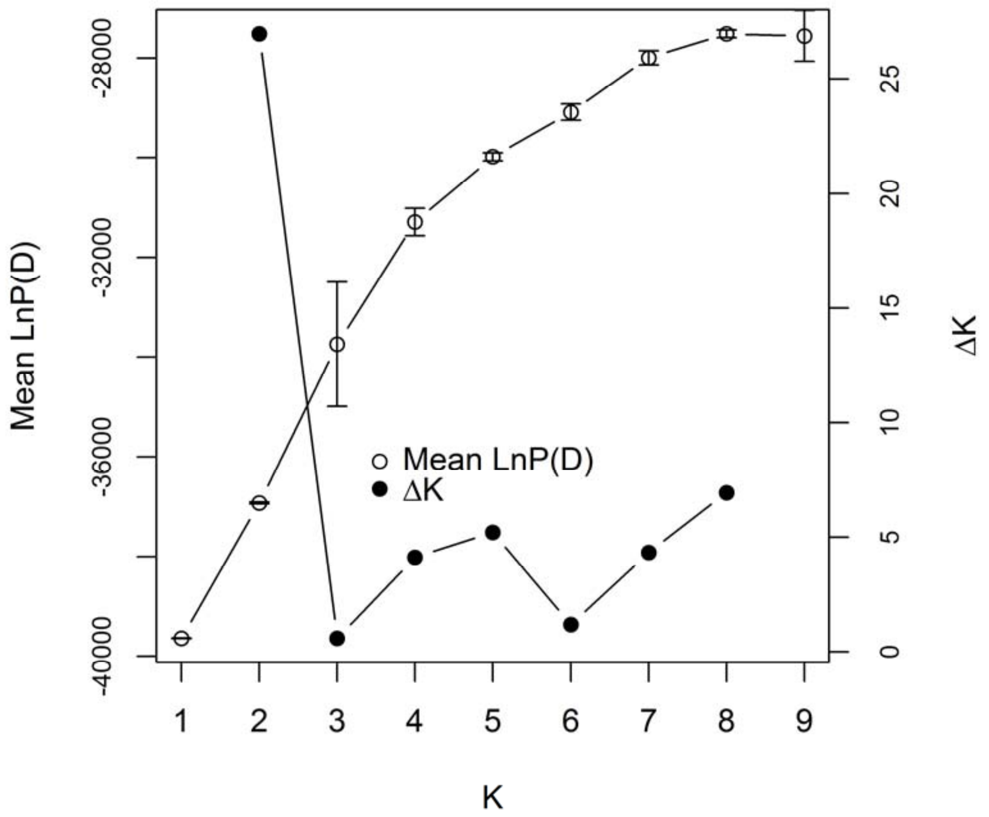

Supplementary Fig. S2. Log likelihood (*Ln*P(D)) and changes in the log likelihood (*ΔK*) for different scenarios of groupings based on 521 amplified fragment length polymorphic loci of *Musa basjoo* var. *formosana.*

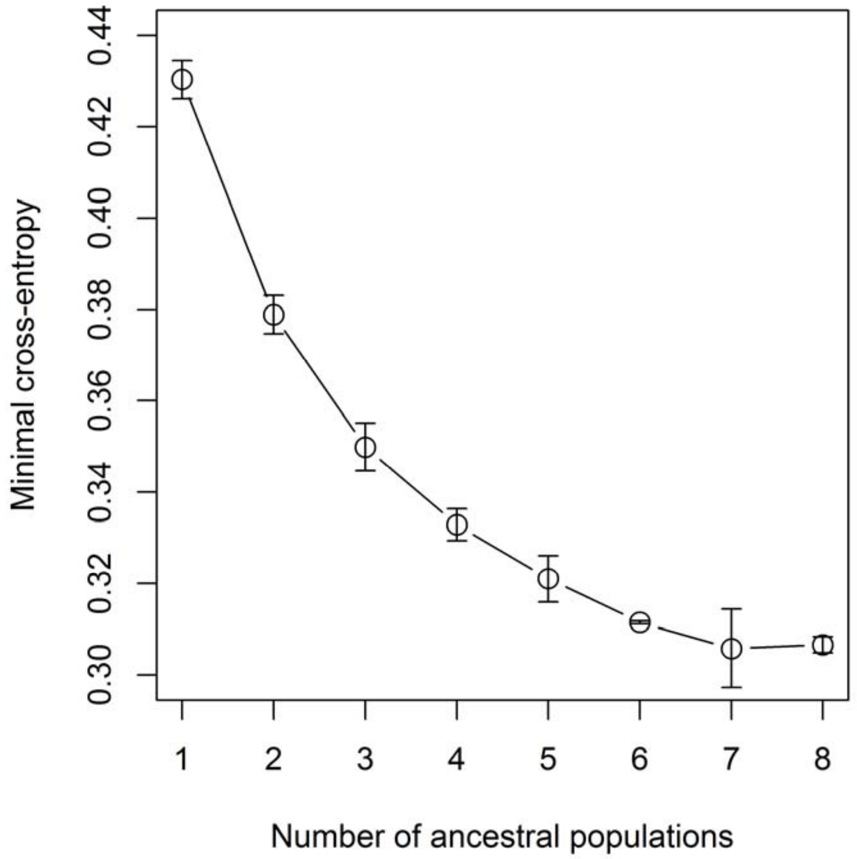

Supplementary Fig. S3. Values of minimal cross-entropy against number of clustering scenario (*K* = 1-8) analszed using the R package "LEA".

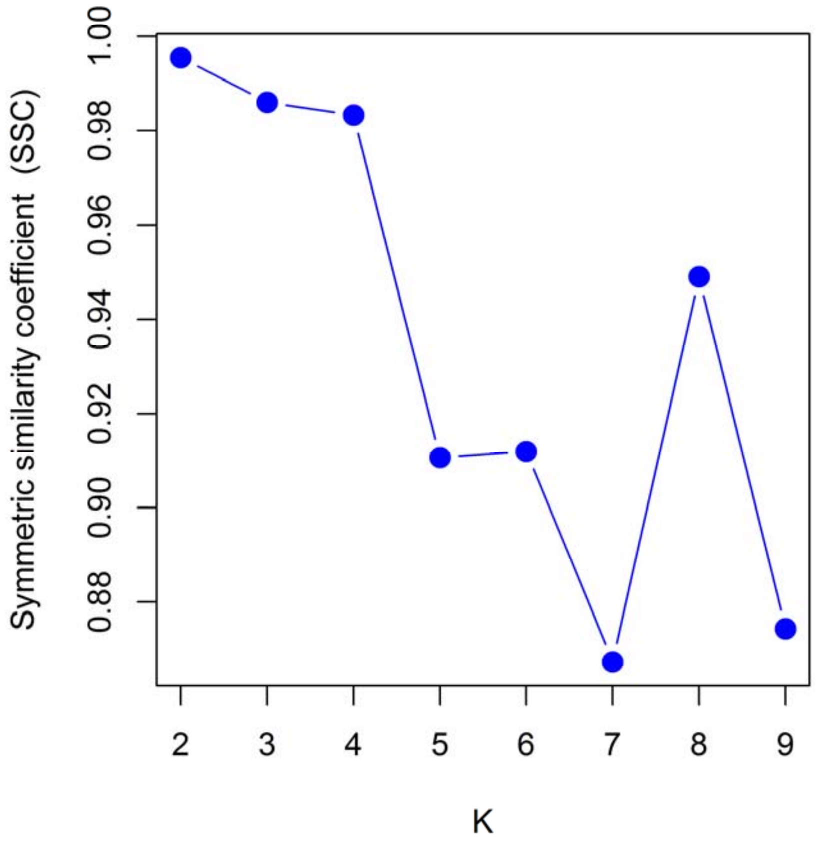

Supplementary Fig. S4. The symmetric similarity coefficient (SSC) for different clustering scenario (*K* = 1-9) [9] summarised using the R package "pophelper".

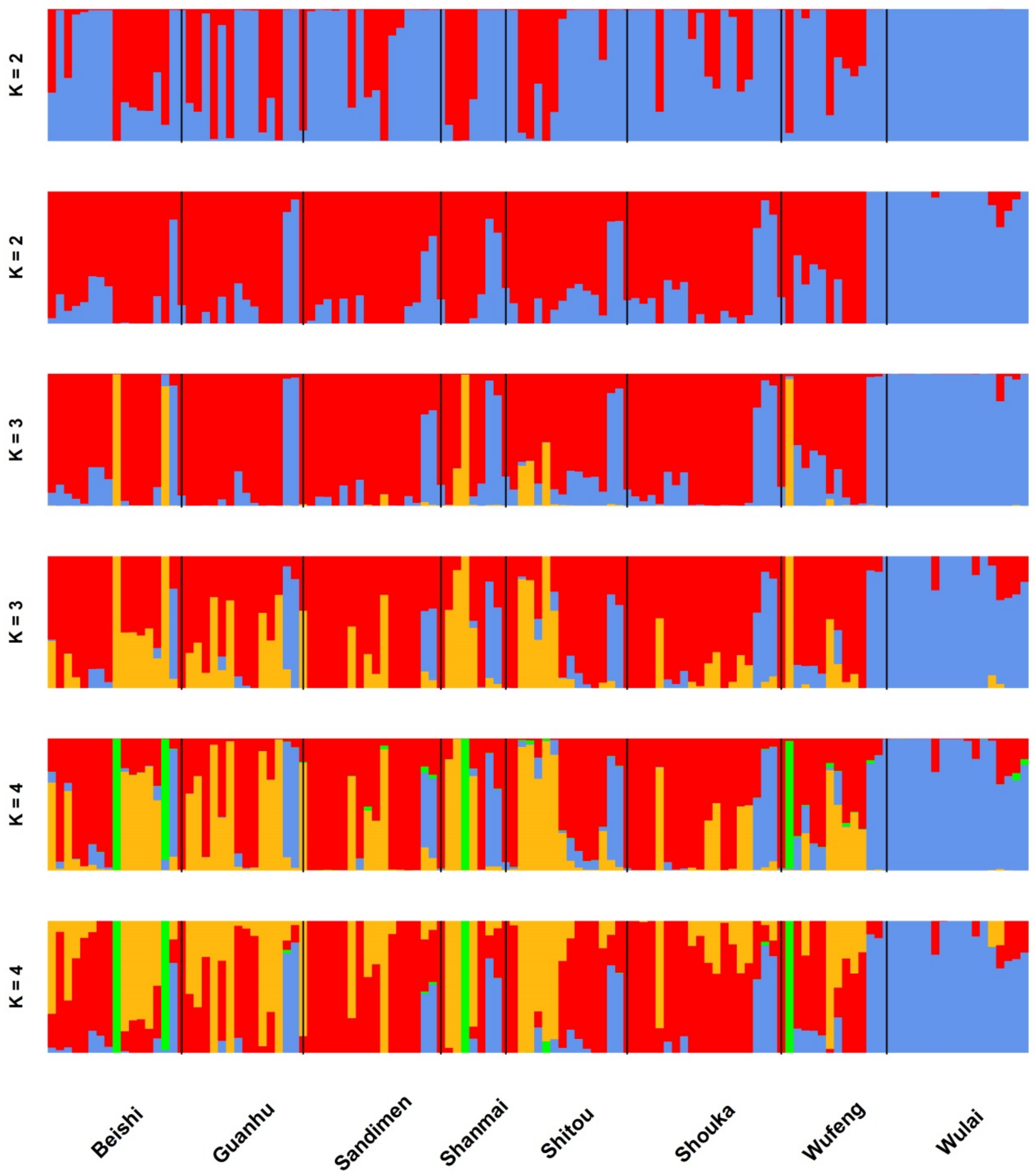

Supplementary Fig. S5. Bar plots represent STRUCTURE (upper panel) and LEA (bottom panel) inference of individual assignments for each K (K = 2-4) based on 521 amplified fragment length polymorphic loci for individuals of eight populations of *Musa basjoo* var. *formosana*.
